# High expression of MORC2 predicts worse neoadjuvant chemotherapy efficacy in triple negative breast cancer

**DOI:** 10.1097/MD.0000000000034164

**Published:** 2023-06-23

**Authors:** Xiaohong Liao, Chao Liu, Zhenluo Ding, Chen Wang, Jing He, Shugui Wu

**Affiliations:** a Department of Oncology, Ganzhou People’s Hospital (The Affiliated Ganzhou Hospital of Nanchang University), Ganzhou, China; b Department of Breast Surgery, Ganzhou People’s Hospital (The Affiliated Ganzhou Hospital of Nanchang University), Ganzhou, China.

**Keywords:** MORC2, neoadjuvant chemotherapy, triple negative breast cancer, tumor-infiltrating lymphocytes

## Abstract

Tumor infiltrating lymphocytes (TILs) are closely related to the patients’ prognosis. Recently, Microrchidia 2 (MORC2) has been documented as a prognostic and predictive biomarker in triple negative breast cancer (TNBC). To compare whether MORC2 is a better predictor than TILs, as well as clinicopathological parameters, in predicting the efficacy of neoadjuvant chemotherapy (NAC) in TNBC, we detected the expression of MORC2 on neoplastic cells through immunohistochemistry and quantified the stromal TILs through Hematoxylin-eosin staining on core biopsies from 50 locally advanced TNBC patients who underwent standard NAC. Among all the 50 patients, 28 (56%) cases had residual tumors, while the other 22 (44%) achieved pathologic complete response (pCR). In these studied patients, age and T-stage showed no correlation with pCR rate, while percentage of TILs, nodal involvement and expression of MORC2 on tumor cells showed significant association with pCR rate. Positive nodal involvement was correlation with worse pathologic response at multivariate analysis (*P* = .0036), and high TILs levels (≥50%) was positively associated with better NAC efficacy at univariate analysis (*P* = .002). Whereas high expression of MORC2 was statistically associated with worse pCR rate both at univariate (*P* < .001) and multivariate (*P* = .036) analysis. Our results indicate that MORC2 expression has a better predictive role in predicting the efficacy of NAC than TILs in TNBC patients.

## 1. Introduction

Triple negative breast cancer (TNBC) is defined by the lack of estrogen receptor, progesterone receptor, and human epidermal growth factor receptor 2, and accounts for 15% to 20% of all invasive breast carcinomas.^[1]^ It is often characterized by earlier age of onset, higher histological grade, higher rates of distant metastasis and local recurrence, presence of lymphocytic infiltration, and worse prognosis as compare to other breast cancer subtypes.^[1,2]^ Currently, for the locally advanced TNBC patients, the combined treatment modalities include surgery resection, radiotherapy and adjuvant or neoadjuvant chemotherapy.^[1,3]^ TNBC is chemotherapy sensitive, and this remains the cornerstone of treatment despite its limited benefit. The current standard of care for newly diagnosed early TNBC consists of neoadjuvant chemotherapy (NAC), followed by surgery. NAC is increasingly used in the management of TNBC, with a pathologic complete response (pCR) rate about 30%, resulting in improved survival.^[4]^ Recently, the addition of immune checkpoint inhibitors to NAC has been reported to significantly improve the pCR rate of TNBC patients.^[5]^ Studies have shown that patients who achieved pCR after NAC tend to have improved disease-free survival and overall survival (OS) compared to patients with residual invasive disease.^[4,6]^ But the serious treatment-related side effects caused by NAC combined with immune checkpoint inhibitors options are difficult to overcome at present. Thus, it is necessary for identifying additional pathological markers to predict the response to NAC and optimize the therapeutic efficacy of TNBC.

TNBC is more immunogenic than other breast cancer subtypes with tumor-infiltrating lymphocytes (TILs) in its microenvironment. Recent studies have demonstrated that the number of stromal TILs is a favorable prognostic factor for improving survival of TNBC patients.^[7,8]^ Increased infiltrating of stromal TILs is significantly associated with prolonged disease-free survival and OS.^[7,9]^ Additionally, it has been well documented that high percentages of TILs represents a predictive biomarker of better NAC efficacy,^[7,9]^ and the high numbers of stromal TILs (≥50%) in TNBC indicates an increase pCR rate to NAC.^[9]^ However, TNBC also shows a high level of programmed cell death-ligand 1 (PD-L1) expression compared with other breast cancer subtypes,^[10,11]^ and immunotherapies targeting the programmed cell death-1 receptor/PD-L1 pathway that maintains immunosuppression in tumor environment in TNBC have been explored and approved for TNBC therapy.^[5,12]^ Thus, the contradictions between TILs and PD-L1/programmed cell death-1 highlights the need for the development of new therapeutic approaches in TNBC.

Recently, the signaling pathway that participates in anti-tumor immune response has been the most popular research topic, and the molecules involved in these pathways have been the most promising targets for anti-tumor therapy. Among these molecules, Microrchidia (MORC) family CW-type zinc finger 2 (MORC2) has been proved as an effective prognostic factor for prognosis in several cancer types.^[13–15]^ MORC2, is a newly identified chromatin remodeling protein with a role in the regulation of DNA damage response, gene transcription, lipogenesis, glucose metabolism and epigenetic regulation.^[15–17]^ MORC2 is demonstrated to be extensively expressed in human cells and has been found to be upregulated in breast cancer, hepatoma, lung carcinoma, ovary cancer, and colorectal cancer.^[16]^ Exogenous expression of MORC2 in tumor cells is associated with tumorigenesis, metastasis and therapy resistance.^[13,15,16,18]^ Recent study demonstrated that the overexpression of MORC2 enhances the recruitment of tumor associated macrophages, the M2 macrophages, to the microenvironment resulting in the immunosuppressive microenvironment of lung cancer.^[14]^ The significant role of MORC2 in breast cancer has been reported as promoting metastasis and enhancing the drug resistance and radiotherapy resistance.^[15,18–20]^ However, the correlation between MORC2 and tumor microenvironment in breast cancer still remains unknown and the role of MORC2 in predicting NAC response of TNBC has not been studied. In our retrospective study, we comprehensively evaluated the role of clinicopathological factors, MORC2 and TILs in predicting the efficacy of NAC in TNBC patients through analyzing 50 pre-NAC core biopsies and comparing the clinicopathological factors, the expression of MORC2 on tumor cells and the percentage of TILs with pCR rate of NAC.

## 2. Materials and methods

### 2.1. The selection of patients

This study is approved by the institutional review board of Ganzhou Peoples’ Hospital. Samples were collected with written informed consent from all patients under Institutional Review Board-approved protocols. All procedures were conducted in accordance with the Declaration of Helsinki and International Ethical Guidelines for Biomedical Research Involving Human Subjects. Between January 2019 and June 2022, 55 locally advanced TNBC patients who underwent standard NAC (4 cycles of epirubixin/doxorubicin + cyclophosphamide every 3 weeks followed by 4 cycles of docetaxel every 3 weeks or 4 cycles of epirubixin/doxorubicin + cyclophosphamide every 3 weeks followed by 12 cycles of paclitaxel weekly or 6 cycles of paclitaxel/docetaxel + epirubixin/doxorubicin + cyclophosphamide). Before starting NAC treatment, each patient’s therapeutic regime was discussed and decided by the multidisciplinary team. Anonymized clinical information, including tumor size, age and the state of nodal involvement at diagnosis, was extracted from data-base of our hospital. After NAC, patients received standard modified-radical mastectomy or breast-conservation surgery with either sentinel lymph node biopsy or axillary dissection based on patients’ condition or individualized requirement. To preliminarily evaluate whether the quantity of neoplastic cells of the pre-NAC core needle biopsies meet the requirements, we obtained the paraffin embedded hematoxylin-eosin (HE) stained sections and calculated the number of tumor cells. Five cases of the 55 patients, with less than 200 tumor cells in HE stained sections, were excluded from this study.

### 2.2. Stromal TILs quantification

To quantify the percentage of the stromal TILs, 2 breast pathologists of our institution reevaluated the HE stained slides from the 50 selected cases blindly, according to the standardized qualitative and quantitative method, which was proposed by the International TILs Working Group 2014.^[21]^ Then we categorized the cases into 3 different subgroups according to the stromal TILs percentage: as 0% to <10%; ≥10% to 49%; and ≥50%. Cases in 0% to <10% and ≥10% to 49% groups were defined as “low TILs,” while cases in ≥50% group were defined as “high TILs.”

### 2.3. Evaluation of MORC2 expression

The expression of MORC2 on tumor cells was evaluated through the additional paraffin-embedded biopsy samples. Immunohistochemistry (IHC) staining was carried out using EnVision Detection System Peroxidase/DAB (DAKO, Santa Clara, CA) following the manufacturer’s recommendations. The primary antibody against human MORC2 (Novus, #NBP1-89295) was diluted at dilution of 1:100 and then incubated at 4°C overnight in a humidified container. Positive and negative controls were used for each run of staining. Interpretation of the IHC results was performed by 2 independent pathologists who were blinded to the clinicopathological information. Slides were evaluated using light microscopy and a standard semiquantitative immunoreactivity score. Whether there is cytoplasmic staining or not, MORC2 was considered to be positive as long as there is nuclear staining, either strong or weak. The percentage of MORC2 was evaluated on all of the neoplastic cells in each biopsy samples and scored as 0% to 100%. The intensity of nucleus staining was recorded through the semi-quantitative methods as absent (0), weak (1), moderate (2), and strong (3). The median value of MORC2 expression was calculated and the specimens were classified into 2 groups: MORC2 expression levels below and equal to the median value as “low-MORC2” and above the median value as “high-MORC2,” respectively.

### 2.4. Evaluation of the pathologic response to NAC

The breast pathologists extensively sampled the surgical specimens based on standardized protocols.^[22,23]^ The pathological stage classification of post-therapy was provided according to the 8th edition of the American Joint Committee on Cancer staging system.^[24]^ A pCR was defined as the clearance of tumor cells in invasive breast cancer tissues and metastatic regional lymph nodes, regardless of the presence of residual ductal carcinoma in site (ypT0/Tis, ypN0).

### 2.5. Statistical analysis

The quantitative variables were presented as mean and range, while the qualitative variables were described as number and percentage. Correlation was analyzed by using Spearman’s rho and comparison was estimated through the nonparametric Mann–Whitney *U* test. The Cohen’s K was applied to detect the consistence between the 2 breast pathologists about the evaluation of TILs and MORC2 expression. Univariable and multivariate logistic regression models were carried out to analyze the clinicopathological factors associated with pCR. Results of both univariate and multivariate analysis were presented as odds ratios and 95% confidence intervals. The *P* value less than .05 was supposed to be statistically significant. All statistical analysis of this study were performed by IBM SPSS Statistics for Windows Version 27.0 (Armonk, NY) or GraphPad Prism (GraphPad, Inc., San Diego, CA).

## 3. Results

### 3.1. Clinicopathological parameters of the cases

The clinicopathological characteristics of the selected patients are summarized in Table 1. Median age at the time of diagnosis was 47 (range from 32 to 62). In 86% of the patients, the diameter of the tumor before NAC was greater than 2 cm. The preliminary assessment of axillary node metastasis was conducted through echography or magnetic resonance and then confirmed by fine needle aspiration cytology or biopsy, was presented in 30 patients (60%). The histologic subtype of all the cases was ductal carcinoma of no special type. According to Elston and Ellis standard, the 33.3% of tumors were high nuclear grade (G3) invasive carcinoma and were poorly differentiated.^[25]^ After NAC, 38 patients (76%) underwent modified-radical mastectomy and 12 (24%) had breast-conservation surgery. The pCR was achieved in 22 patients (44%).

**Table 1:** Clinico-pathological features of the study population.

Characteristic	N (%)	Complete response N = 22 (44%)	Incomplete response N = 28 (56%)
Age, yr			
<50	27 (54)	12 (44)	15 (56)
50+	23 (46)	10 (43)	13 (57)
T-stage			
cT1	7 (14)	5 (71)	2 (29)
cT2	32 (64)	13 (41)	19 (59)
cT3	7 (14)	3 (43)	4 (57)
cT4	4 (8)	1 (25)	3 (75)
Nodal involvement			
cN0	20 (40)	12 (60)	8 (40)
cN+	30 (60)	10 (33)	20 (67)
Surgery			
Mastectomy	38 (76)	14 (37)	24 (63)
Conservative surgery	12 (24)	8 (67)	4 (33)
MORC2			
≤Median	28 (56)	19 (68)	9 (32)
>Median	22 (44)	3 (14)	19 (86)
TILs			
0–10%	15 (30)	4 (27)	11 (73)
≥10–49%	20 (40)	6 (30)	14 (70)
≥50%	15 (30)	12 (80)	3 (20)

MORC2 = MORC family CW-type zinc finger 2, TILs = tumor-infiltrating lymphocytes.

### 3.2. Percentage of stromal TILs

**Figure 1.**
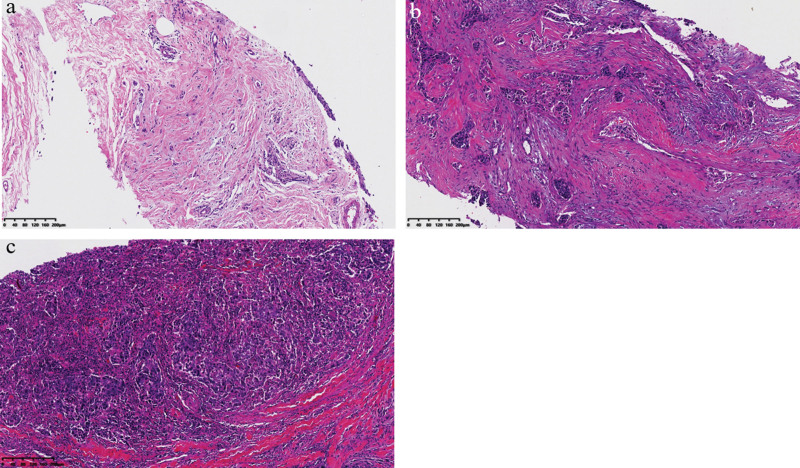
Different stromal TILs categories in TNBC core biopsies. Representative pictures of low TILs (A), intermediate TILs (B), and high TILs (C). Hematoxylin-eosin, original magnification ×10. TILs = tumor infiltrating lymphocytes, TNBC = triple negative breast cancer.

Stromal TILs were detected in the majority of pre-NAC biopsy samples (n = 44; 88%) and the percentages of TILs was recorded ranging from 2% to 75% (Fig. 1). High TILs, with the definition of ≥50% of the tumor stroma, were found in 15/50 cases (30%). The kappa statistic for TILs estimation was 0.58 (standard error, 0.04).

### 3.3. Expression of MORC2 on neoplastic cells

**Figure 2.**
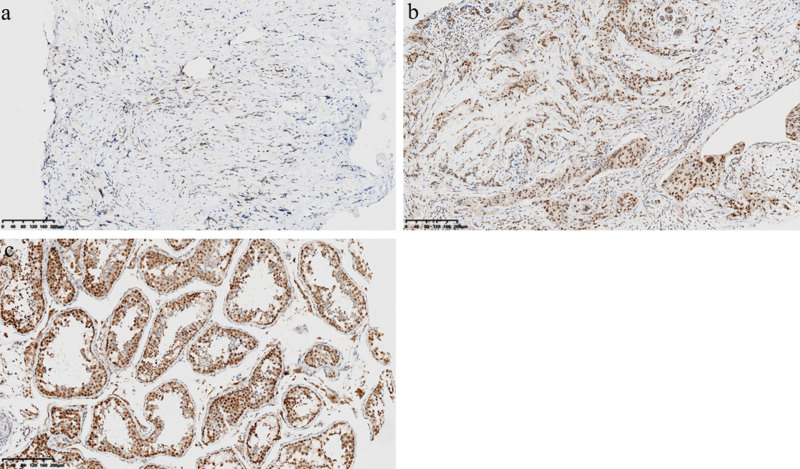
Staining intensity of MORC2 on neoplastic cells in TNBC core biopsies. MORC2 immunohistochemical stain on neoplastic cells, scored as weak (1+, A), moderate (2+, B), and strong (3+, C). MORC2 = microrchidia 2, TNBC = triple negative breast cancer.

The median expression value of MORC2 on neoplastic cells was 50% (range 0–95%). Levels below or equal to this value were classified as “low MORC2” and above the value were defined as “high MORC2.” Twenty-two cases (44%) were “high MORC2” with a mean score of 75.2%; in the “low MORC2” group, the mean expression value was 20.7%. The staining intensity was scored respectively as 1 + 22%, 2+ in 36%, 3+ in 42% of the core biopsy samples (Fig. 2). The immunostaining absence of MORC2 on neoplastic cells was recorded in 3 out of 50 pre-NAC biopsies (6%). The kappa statistic for MORC2 evaluation was 0.72 (standard error, 0.05).

### 3.4. Correlation of clinicopathological characteristics with NAC efficacy

The association between pCR rate and clinicopathological characteristics, including age, T-stage, nodal involvement, MORC2 expression and percentage of TILs, were analyzed in the studied patients. Age and T-stage showed no correlation with pCR rate, while percentage of TILs, nodal involvement and expression of MORC2 on neoplastic cells showed significant association with pCR. The pCR rate of patients with positive node metastasis (33%) was much lower than that of negative ones (60%) (Table 1). Positive nodal involvement was correlation with worse pathologic response in multivariate analysis (*P* = .0036) (Table 2). The pCR to NAC in high TILs group was achieved in 80% of patients, while only 27% in low TILs group in pre-NAC biopsy (Table 1). Levels of TILs were associated with a better response in univariate analysis (*P* = .002) (Table 2). However, low levels of MORC2 on neoplastic cells were correlated with higher pCR rate (Table 1). Levels of MORC2 were associated with pCR both at univariate (*P* < .001) and multivariate (*P* = .036) analysis (Table 2). There was no significant correlation between the rate of pCR and the staining intensity of MORC2 on neoplastic cells.

**Table 2:** Univariate and multivariate analysis of factors associated with pCR.

Characteristic	Univariate analysis		Multivariate analysis	
	*P* value	Odds ratio (95% CI)	*P* value	Odds ratio (95% CI)
Age				
<50	.945	1.04 (0.339–3.19)	.839	1.179 (0.24–5.784)
≥50				
T-stage				
cT1	.455	2.053 (0.192–21.97)	.412	1.89 (0.075–47.479)
cT2				
cT3				
cT4				
Nodal involvement				
cN0	.066	3 (0.928–9.697)	.036*	8.885 (1.148–68.781)
cN+				
MORC2				
≤Median	<.001*	13.37 (3.126–57.18)	.036*	6.885 (1.136–41.726)
>Median				
TILs				
<10%	.002*	0.1 (0.023–0.4321)	.184	0.26 (0.036–1.9)
≥10% to <50%				
≥50%				

CI = confidence intervals, MORC2 = MORC family CW-type zinc finger 2, pCR = pathologic complete response, TILs = tumor-infiltrating lymphocytes.

* indicates *P*<0.05.

## 4. Discussion

In recent years, studies have shown that the administration of NAC in TNBC has a significantly higher pathological remission and can improve the prognosis of TNBC patients.^[3,4,6]^ However, the pCR rates of standard NAC is slightly over 30% of cases. With the addition of immunotherapy to NAC in TNBC patients, the pCR rate increases to over 65%.^[5]^ But the serious treatment-related side effects of these options limit the number of available patients. Thus, it would be advisable to identify predictive biomarkers to guide the choice of the most appropriate therapy regime for TNBC patients in neoadjuvant setting. The mechanisms of resistance to NAC could involve in cancer cells and tumor microenvironment.^[26]^ Tumor microenvironment has been increasingly demonstrated as a major regulator of tumorigenesis and drug resistance.^[27,28]^ A growing number of studies have focused on identifying biomarkers in tumor microenvironment to predict clinical outcome and guide therapy in TNBC.^[7–9]^ The stromal tumor infiltrating immune cells, especially the TILs, has been documented as the prognostic indicator in predicting response to therapy in TNBC.^[29,30]^ However, studies in the literature have mainly focused on the percentage of TILs, and the significant differences in predicting response to therapy are only shown in univariate analysis or multivariate analysis.^[30,31]^ For this reason, TILs may not be a good predictor sometimes, and adding new biomarker might improve the assessment.

A novel biomarker of interest is MORC2. Recent study has shown that MORC2 is a critical regulating molecule of tumor microenvironment in immunosuppressive and pro-angiogenesis.^[14]^ MORC2 overexpression was associated with unfavorable pathological conditions, therapy resistance, and poor OS in breast cancer.^[16,32]^ However, the role of MORC2 in predicting NAC response and the relationship between MORC2 and pathologic response of TNBC have not been studied. In this retrospective study, we comprehensively examined patients’ clinicopathological characteristics to identify parameters which can predict the response to NAC in TNBC. Potentially, the most novel finding in this study was that, for the first time, we introduced MORC2 to assess the therapy response. We detected MORC2 expression by IHC and found that high expression of MORC2 in tumor cells in pre-NAC biopsies, not only in univariate analysis but also in multivariate analysis, is significantly associated with a lower pCR rate in TNBC patients. We also validated the correlation of high stromal TILs with higher pCR rate in TNBC in univariate analysis, which is consistent with previous observations.^[29,31]^ Thus, its suggestive that MORC2 outperformed TILs in predicting tumor response to NAC in TNBC patients in our data set. Deeper mechanistic study might be needed to further reveal the association between MORC2 and chemotherapy in TNBC.

The limitations of our study include a relatively small sample size and small core tissues. This may explain some discrepancies of our results with some previous studies. Previous studies have demonstrated a negative correlation between nodal involvement and pCR rate after NAC both in univariate analysis and multivariate analysis,^[33]^ suggesting the predictive role of nodal status in response to NAC. In our study, the nodal involvement was significantly associated with pCR in multivariate analysis, but not in univariate analysis. More samples should be included in future studies.

## 5. Conclusions

In summary, we retrospectively analyzed 50 TNBC patients by comparing clinicopathological factors and found MORC2 was strongly associated with pCR. Our results make significant contribution to the search of biomarker, which could be able to select TNBC patients more probable to achieve pCR to standard NAC.

## Acknowledgments

We sincerely acknowledge the staff members of the department of pathology (Ganzhou People’s Hospital) for their excellent technical assistance.

## Author contributions

**Conceptualization:** Xiaohong Liao.

**Formal analysis:** Chao Liu, Zhenluo Ding, Shugui Wu.

**Software:** Shugui Wu.

**Supervision:** Xiaohong Liao, Chen Wang.

**Writing – original draft:** Xiaohong Liao, Chao Liu, Chen Wang, Jing He.

**Writing – review & editing:** Xiaohong Liao, Chao Liu, Chen Wang, Jing He.
